# Expression, Purification and Characterization of a Recombinant Plasmodium Vivax Thrombospondin Related Adhesive Protein (*Pv*TRAP)

**Published:** 2006-09

**Authors:** Jamiu A. Ogunbanwo, Prakash Rao Pendyala, Pawan Malhotra, Virander S. Chauhan

**Affiliations:** 1 *Monash University, School of Biological Sciences, Victoria 3800, Australia;*; 2 *St. Mary’s College, University of Melbourne, Parkville, Victoria, Australia;*; 3 *Department of Molecular Biology, University of Central Florida, Orlando Fl, USA;*; 4 *International Center for Genetic Engineering and Biotechnology, Aruna Asaf Ali Marg, New Delhi, India*

**Keywords:** thrombospondin-related adhesive protein (TRAP), *plasmodium vivax*, malaria

## Abstract

Thrombospondin Related Adhesive Protein (TRAP) is a transmembrane parasite molecule responsible in sporozoite-host interactions. This molecule is one of the most promising vaccine candidates against the pre-erythrocytic forms of malaria. In the present study, a gene encoding the *Plasmodium vivax* TRAP (*Pv*TRAP) was expressed in Escherichia coli (M15 strain) using the expression plasmid pQE30. The expressed recombinant protein *Pv*TRAP of about 70kDa was achieved, purified and refolded according to the standardized refolding procedure. This refolded protein (*Pv*TRAP) showed a single band monomeric form with SDS-PAGE and blot analysis. In reduced and alkylated form, *Pv*TRAP showed less binding to hepatoma (HepG2) liver cells, when compared to the normal purified and refolded form. Purified and refolded recombinant *Pv*TRAP bound Duffy-positive human erythrocytes, while no binding was observed with Duffy-negative erythrocytes. Our report on *Pv*TRAP is currently documented for the first time and it has been able to provide an experimental evidence of the biochemical and binding properties of *Pv*TRAP in the invasion of hepatocytes and interaction with Duffy-positive and Duffy-negative human erythrocytes. In conclusion, our findings have been able to demonstrate the potential of *Pv*TRAP as a promising target for vivax malaria vaccine candidate.

## INTRODUCTION

The reported global human malaria stands at 300-500 million clinical cases with an average of about 2 million deaths per year (1). *Plasmodium falciparum* causes 80% of human malaria’s morbidity and mortality, mostly in sub-Saharan Africa, however, *Plasmodium vivax* annually accounts for 70-80 million cases across much of the tropics and subtropics of the world (1) *Plasmodium vivax* is the second most prevalent species and widely distributed human parasite that causes malaria morbidity among people of all ages in Africa, Asia, the Middle East and Latin America (1). Several antigens expressed at different stages of the parasite life cycle have been characterized and found to have the potential for use in a sub unit vaccine against *P. vivax* (2-5, 43-45).

One of the antigens thrombospondin-related adhesive protein (TRAP), a potential malaria vaccine candidate, is one of the two major proteins identified on the sporozoite surface of *Plasmodium* species that are involved in hepatocyte or HepG2 cell line recognition / or invasion (6-11). The genes encoding TRAP and the circumsporozoite TRAP related protein (CTRP) are differentially expressed in sporozoites and ookinetes, respectively, two motile forms of *Plasmodium* species found exclusively during the life cycle of the parasite in the mosquito (6, 12-14).

**Figure 1:**
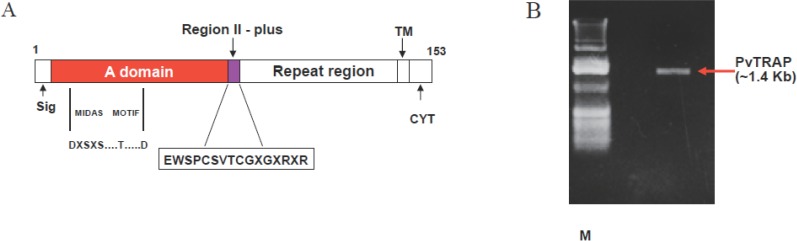
(A) Schematic representation of the gene encoding *Plasmodium vivax* Thrombospondin Related Adhesive Protein (*Pv*TRAP). A domain contains conserved regions of ~200 amino acids. Region II-plus is homologous to Circumsporozoite Surface Protein (CSP) type (A sequence of Plasmodium TRAP is shown). TM-Transmembrane domain, CYT- Highly conserved cytoplasmic tail, Sig-Signal sequence region. Repeat region is rich in asparagine/proline, but not conserved among different TRAP of Plasmodium spp. A full length gene of *Pv*TRAP shown consists of 1530 amino-nucleotide bases in the ratio of 481a; 347c; 376g and 326t. (B) Cloned and expressed nearly full length *Pv*TRAP gene (~1.4 Kb), excluding signal peptide sequence, transmembrane domain (TM) and highly conserved cytoplasmic tail (CYT).

TRAP is found in the micronemes and a type 1 transmembrane protein (Fig. 1) whose ectodomain consists of (i) an A domain, (ii) a TSR, and (iii) a repeat region of variable length and sequence, depending on the plasmodial species. The A domain is a ~ 200 residue -long adhesive module that was first recognized in the plasma protein von Willebrand factor (15). It now defines a superfamily of soluble proteins, including complement protein factor B and C2, extra cellular matrix proteins, including numerous types of non fibrillar and FACIT (fibril associated collagen with interrupted triple helix) collagens, and integral membrane proteins, including seven integrin and chains (6, 15).

The crystal structures of the A domains of TRAP have been determined (16-19). They comprise alternating amphiphatic and helices and hydrophobic B strands conforming to the classic and B “Rossmann” fold (15). Identification of 5 residues which coordinate a divalent cation (Mg^2+^ or Mn^2+^) in A domains, define the metal-ion dependent adhesion site (MIDAS) motif (Fig. 1a), which is conserved in a number of A domains of TRAP (15). Mutational analysis of the metal ion-dependent adhesion site motif in integrin-a chain has demonstrated the critical role of these five residues in ligand-binding (20-24).

In human living in malaria endemic areas, reports have shown close correlation between levels of TRAP antibodies and clinical protection against malaria (25). Also, a Babesia bovis merozoite protein was discovered to have domain architecture highly homologous to *Plasmodium falciparum* TRAP (45). Antisera to the TSR region of TRAP inhibits the *in vitro* invasion of erythrocytes by the asexual blood stage merozoites and also recognizes a TRAP-like protein in the blood stage lysate of *P. falciparum* (26). Therefore the possible role of TRAP in two most important different stages of the parasite: the sporozoite invasion of hepatocytes and merozoite invasion of erythrocytes makes the molecule a potential malaria vaccine candidate.

We have expressed *Pv*TRAP nearly full length gene in *Escherichia coli*, purified and refolded the protein with established standard procedures and characterized the protein to ascertain its biological and functional activities as a potential target for the development of a protective and safe vivax malaria vaccine.

## MATERIALS AND METHODS

### Amplification and Cloning Strategy for *Pv*TRAP gene

We obtained blood from an Indian *P. vivax* infected patient by venipuncture and passed through CF-11 column (Whatman) to remove leukocytes. The parasitized erythrocytes were purified by centrifugation on a ficoll-hypaque gradient and subjected to saponin lysis to get a sporozoite rich preparation. DNA was isolated from the sporozoite rich preparation by standard procedures (27). Based on sequence comparison of *Pv*TRAP with *Pk*TRAP and *Pg*TRAP (9) a set of primers from the conserved sequences, one near the putative signal peptide cleavage site, forward primer.

(5’-GCGGATCCGACGAAATAAAGTATAGTGAAGAAGTATG-3’) and the other at the extreme carboxyl-terminus, a reverse primer (5’-CACTCAAGCTTAAATTTTGTAGCCATTATT-3’) were synthesized.

PCR amplification was achieved using 100 ng of *P. vivax* genomic DNA in steps, with initial hot start of 94°C/3 min followed by 5 cycles of 94°C/1 min, 44°C/2 min and 72°C/3 min and 25 cycles of 94°C/50s, 48°C/1 min and 72°C/3 min.

A fragment of about 1.4 Kb was amplified from *P. vivax* genomic DNA. The PCR product was gel purified using QIA quick gel extraction kit (QIAGEN) and cloned into the pGEM-T cloning vector (Promega) as per the manufacturer’s instructions.

Positive clones were selected by Southern hybridization and restriction analysis sequencing was performed using the dideaxy chain termination method (Sequenase, USB), with vector specific and gene specific sequencing primers. Eight independent clones were sequenced to rule out the possibility of any PCR generated artifacts. Sequence analysis and alignment were carried out using MAC-VECTOR and DNASTAR (data not shown).

### Expression, purification and refolding of recombinant *Pv*TRAP

The PCR fragment of about 1.4 Kb full lengths *Pv*TRAP gene was digested with Bam HI and Hind III and then sub-cloned into pQE-30 plasmid vector which had been digested with the same restriction enzymes. The resulting ligation product- pQE-30-His tagged *Pv*TRAP was used to transform the *E. coli* (M15) cells. Ampicillin and Kanamycin resistant clones containing pQE30-His-6-*Pv*TRAP plasmid were identified by restriction with Bam H1 and Hind 111 (Fig. 1b). Competent *E. coli* M15 cells were transformed with plasmid pQE-30-His-6-*Pv*TRAP and transformants were selected on LB medium containing Ampicillin (100 μg/ml) and Kanamycin (25 μg/ml). A single colony was inoculated into 50 ml of LB medium containing the same antibiotics and grown at 37°C; 200 r.p.m. until OD600 reached about 0.6-0.7. An aliquot (5ml) of this pre-culture was transferred into 1 liter LB medium containing the same antibiotics, incubated on a shaker at 37°C; 200 r.p.m. for 2-4 hr. The protein expression was induced by addition of isopropyl-β-D-thiogalactopyranoside (IPTG) to a final concentration of 1mM when OD600 reached 0.6-0.7.

After a 6-8 hr post incubation period, the bacterial cells were harvested by centrifugation at 5000g at 4°C for 45 min. The induced and uninduced expressed *Pv*TRAP was comparatively analyzed on SDS-PAGE (data not shown). The cell pellet obtained after harvesting the bacterial cells (*E. coli*) was suspended in ice-cold TBS (20 mMTris-Cl, pH7.5, 250 mM NaCl).

The washed *E. coli* cell pellet containing *Pv*TRAP (insoluble protein in inclusion bodies) was thawed in ice and solubilized in lysis buffer (6 MGu.HCl, 20 mMTris-Cl, pH8.0, 250 mM NaCl; 5 mL/g wet weight of the pellet) at room temperature for 60 minutes. After centrifugation (10,000 × g 30 min. 4°C), the supernatant was incubated with Nickel-Nitriloacetate acid (Ni-NTA) agarose resin (1.0 ml of 50% slurry was used for the lysate from 2 g of the induced bacteria cell pellet) which was already equilibrated with the lysis buffer for 1 hr at room temperature.

The resin-lysate suspension was poured to a Bio-Rad-Poly-Prep Chromatography column (1.5 × 12 cm propylene column) and the unbound proteins were allowed to pass down. Nickel- Nitriloacetate acid (Ni-NTA) agarose resin with bound proteins was washed with (a) lysis buffer (20 × bed volume), (b) 10 × bed volume of wash buffer 1 (8 M Urea, 20 mM Tris-Cl, pH 8.0, 250 mM NaCl) and (c) 10 × bed volume of wash buffer 2 (6M Urea, 20 mMTris-Cl, pH8.0, 250 mM NaCl).

After washing, the bound *Pv*TRAP was eluted successfully with 100 mM imidazole in 6M Urea, 20mM Tris-Cl, pH8.0.

**Figure 2:**
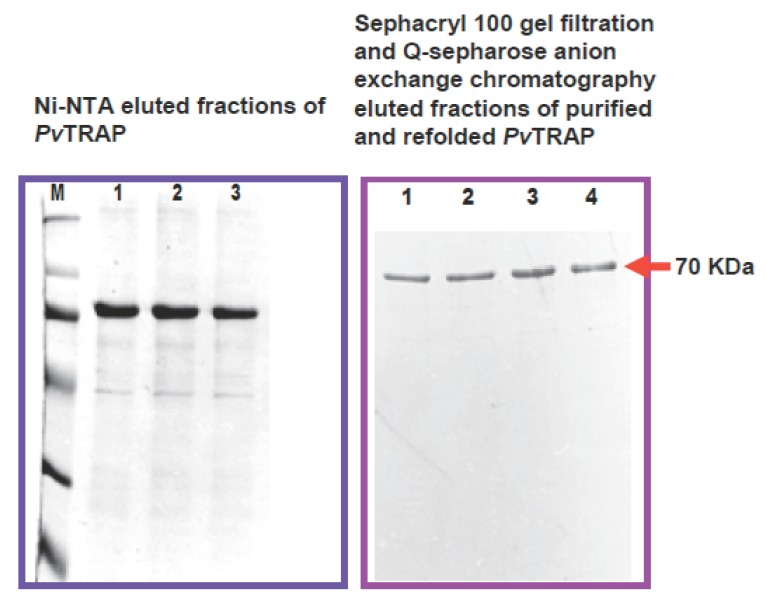
Expression, purification and refolding of recombinant *Pv*TRAP. Left panel shows the position of the molecular – mass markers (in kDa), top labels 1, 2, 3 indicate the eluted fractions of *Pv*TRAP purified by Ni-NTA chromatography. The right panel with top labels 1, 2, 3, 4 clearly indicates the eluted fractions of refolded protein further purified by Sephacryl (S-100) gel filtration and Q-sepharose anion-exchange chromatography. The purified and refolded recombinant *Pv*TRAP was resolved on SDS-PAGE under reducing conditions on a 10% gel stained with Coomasie Blue. The protein *Pv*TRAP was shown to occupy ~70 kDa molecular mass as shown on the right of the left panel.

The eluted *Pv*TRAP was further purified by anion exchange chromatography (Q-Sepharose; Pharmacia) under denaturing condition. The anion exchange chromatography was performed by using Fast Performance Liquid Chromatography (FPLC) system (Pharmacia) (Buffer A: 6 M Urea, 20 mM Tris-Cl, pH 8.0; Buffer B: 6 M Urea, 20 mMTris-Cl, pH8.0 and 1 M NaCl), at 0.75 ml/min flow rate. Elution of the purified *Pv*TRAP was carried out with increasing concentration of salt (30-40% of Buffer B in about 30 min) and monitored at 280 nm. 1mL elution fractions corresponding to the peak were collected and analyzed by SDS-PAGE for purity. Protein concentration was determined by the method of Bradford (28) using bovine serum albumin as a reference. Protein SDS-polyacrylamide gel was performed by the method described by Chang *et al*. (29). Further purification with Sephacryl 100 (S-100) gel filtration was carried out in order to achieve a single band purified protein (Fig. 2). A standardized *in vitro* refolding procedure was designed and employed for the protein (*Pv*TRAP) with modification of the method described by Glansbeek *et al*. (30).

The purified *Pv*TRAP was rapidly diluted in various buffers containing 20 mM Tris-Cl pH8.6, 2 mM EDTA, 20 mM NaCl, 10% Glycerol, 2 mM Glutathione (reduced-GSH) and 0.2 mM oxidized Glutathione-(GSSG) and the refolding was carried out at the temperature 10°C within the duration of 40-70 h.

The refolded *Pv*TRAP was concentrated and dialyzed against 200 mM Tris pH7.5, 200 mM NaCl and 1% glycerol at 4°C for 4 h. The final dialysis was carried out with phosphate buffered saline (PBS) pH7.2 overnight at 4°C. The protein (*Pv*TRAP) was refolded immediately after Ni-NTA purification laboratory procedure was carried out.

### Characterization of recombinant *Pv*TRAP

**Circular Dichroism of recombinant *Pv*TRAP.** Circular dichroism (CD) spectra of refolded *Pv*TRAP were recorded on a Jasco model J-720 spectropolarimeter at 50-100 μg/ml protein in 10 mM sodium phosphate pH7.0. All measurements were carried out in a 0.1-mm path length cylindrical cuvette at room temperature at 180-260nm. The number of spectra was recorded at a scan speed of 10nm/min with a step resolution of about 0.1nm (40) (data not shown).

**Reduction and Alkylation of recombinant *Pv*TRAP.** By modification of the procedure described by Aitkin and Learmonth (the protein biochemistry protocols), the refolded *Pv*TRAP was reduced with DTT (0.02 M final concentration) under N2 for 2 hr at 45°C. The reduced *Pv*TRAP protein was alkylated (in dark) with 0.1 M iodoacetamide with stirring at 37°C for 1 hr. This reduced and alkylated *Pv*TRAP was desalted on prepared Sephadex 25 column and estimated by Bradford (BioRad) assay accordingly (31).

**Figure 3:**
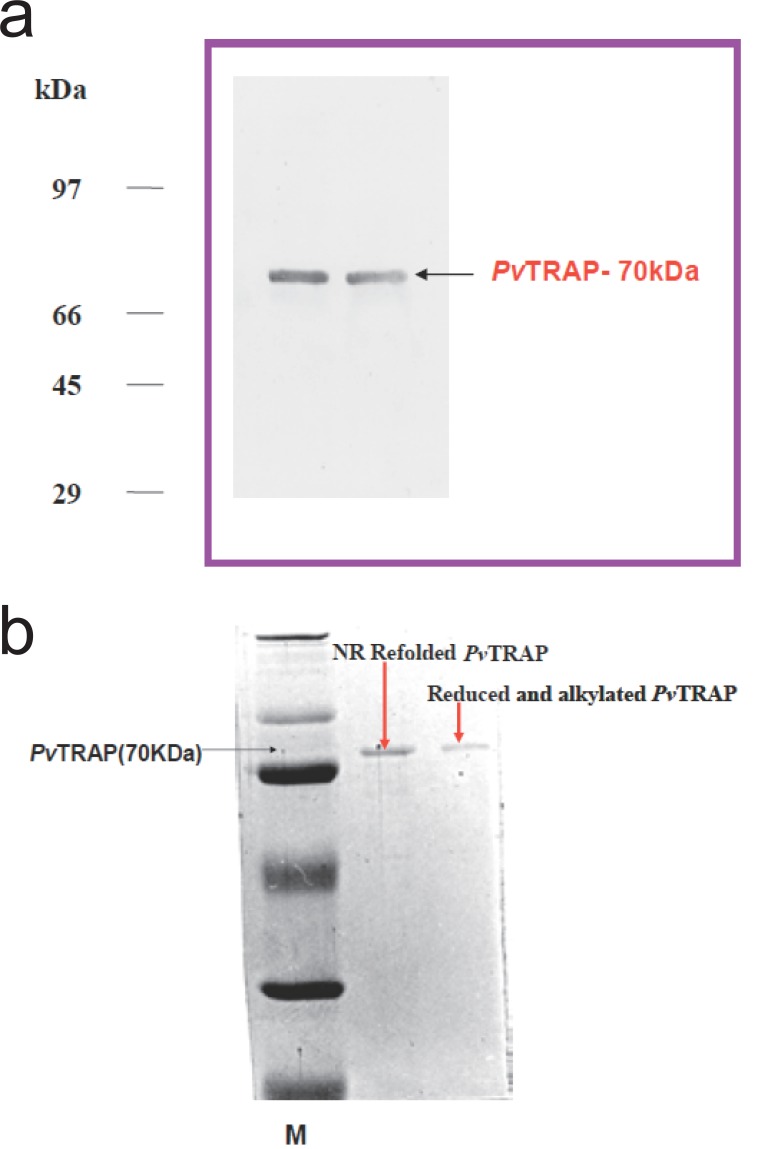
**a**. Immunoblot analysis of rabbit polyclonal antiserum against recombinant purified and refolded *Pv*TRAP construct. The protein *Pv*TRAP was recognized by polyclonal antibodies raised in rabbit (serum dilution 1: 10,000). The pattern of migration of molecular weight standards (Sigma) is shown on the left side of the panel in kDa; **b**. Immunoblot analysis of rabbit polyclonal antiserum against recombinant purified and refolded protein *Pv*TRAP treated under different biochemical conditions. The protein was treated as reduced and alkylated *Pv*TRAP, non-reduced (NR) refolded *Pv*TRAP as shown. The protein *Pv*TRAP was resolved by SDS-PAGE under the treatment conditions on 10% gels, transferred on to *Pv*DF membranes. The membranes were probed with the polyclonal antibodies raised in rabbit and directed against *Pv*TRAP. The molecular mass marker (M) is shown at the left side of the panel.

*Pv*TRAP purified and refolded samples were analyzed by discontinuous SDS-PAGE (29), each sample was denatured in a sample buffer with or without β-Mercapto-ethanol by boiling at 96°C for 10 min and then used for electrophoresis on 10% SDS/polyacrylamide gel. Protein staining was performed with Coomassie brilliant blue (Fig. 3b).

**Immunoblotting.** For immunologic characterization, anti *Pv*TRAP sera were raised in rabbit and generated in our laboratory for immunoblot assay (32). The purified and refolded *Pv*TRAP was transferred from the unstained SDS gel to a nitro-cellulose system (Biorad) at 990 mA for 2 hr. After blocking with 5% milk in phosphate Buffer Saline PBS containing 0.01% Tween 20 (buffer A), the membrane was incubated with anti-*Pv*TRAP antibodies (1:10,000) raised in rabbit at room temperature for 1 hr. Unbound antibodies were removed by intensive washing in buffer A and incubated with the secondary horse-radish peroxidase conjugate Anti-IgG specific antibody (Sigma) raised in goat. After repeated washing the labeled *Pv*TRAP was detected by colourizing with appropriate substrate – Diaminobenzidine (DAB) (Sigma) for the conjugated secondary antibody (Fig. 3a & b). *Plasmodium falciparum* TRAP (*Pf*TRAP) and *Plasmodium cynomolgi* (*Pc*TRAP) were used as positive controls to study the cross reactivity with generated anti *Pv*TRAP antibodies (32, 46).

**Hepatoma (HepG2) Liver Cells assay.** Further characterization of *Pv*TRAP was carried out with HepG2 liver cells *in-vitro*. The maintenance of HepG2 liver cells (American Type Culture Collection, Rockville, MD, USA) was carried out with Dulbecco Minimum Essential Medium (DMEM) supplemented with 10% Fetal Calf Serum (FCS). The cells were removed from culture flasks with Trypsin-EDTA (0.25% Trypsin, 1mM EDTA in HBSS) (GIBCO-BRL) and pelleted at 3000 × g for 15 min at 4°C. The cell pellet was resuspended in complete DMEM medium to a concentration of 106 cells/ml. 105 cells/well were plated in 96 well cell culture plates and allowed to grow overnight. The cells were washed with the medium twice at the interval of 10 min and fixed with 4% Para formaldehyde for 15-20 min.

Following washings with TBS (50 mM Tris, ph7.4, 130 mM NaCl), the wells were blocked with 1% BSA in Tris buffer saline (TBS) for 2 hr at 37°C. The cells were incubated with different concentrations of the purified and refolded *Pv*TRAP and/or reduced-alkylated purified and refolded *Pv*TRAP for 1 hr at 37°C.

**Figure 4:**
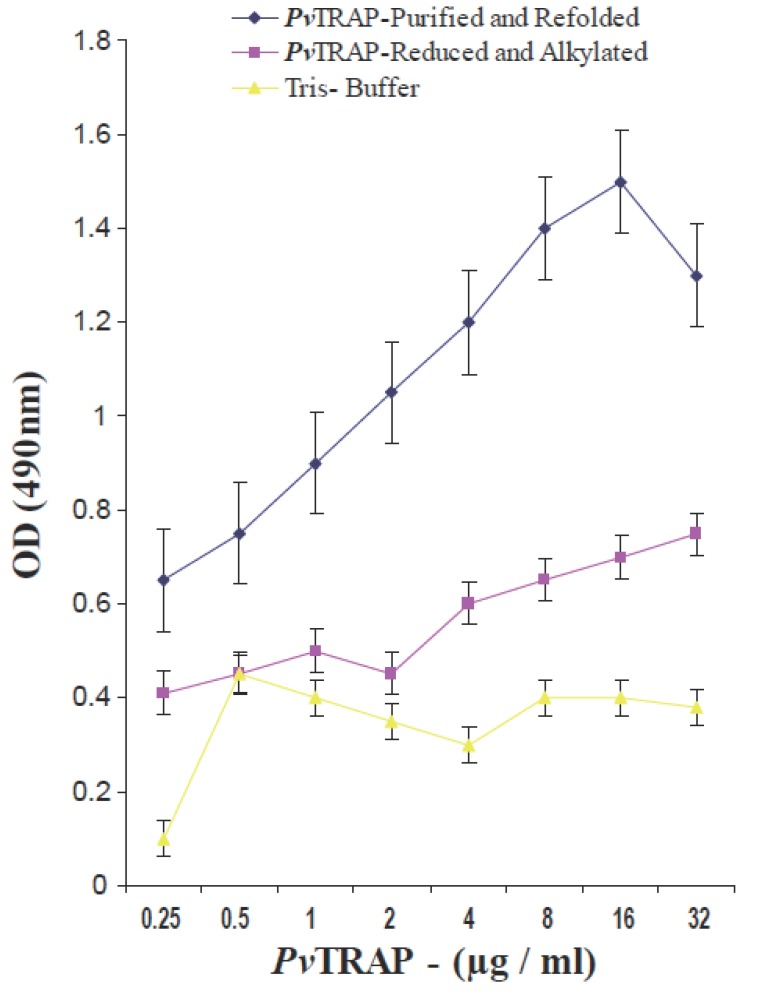
Biochemical characterization of *Pv*TRAP. The specificity and binding of *Pv*TRAP protein to hepatoma (HepG2) cells under different biochemical conditions were shown. The highest binding was observed with recombinant purified and refolded *Pv*TRAP under non-reduced condition. Low binding capacity was observed with the same protein under reduced and alkylated conditions as shown in Figure 4. Reduced and alkylated *Pv*TRAP and Tris Buffer served as controls.

Unbound proteins were removed by washing with TBS three times for 10 min each and incubated with polyclonal antibodies raised in mice against purified and refolded *Pv*TRAP for 45 min at room temperature. The bound antibodies to *Pv*TRAP were detected by goat anti-mouse IgG-horseradish peroxidase (HRP) conjugate (Sigma). Binding of *Pv*TRAP to HepG2 cells was measured at 490 nm using micro plate reader (Molecular Devices–USA). Absorbance obtained at 490 nm was plotted against different concentrations of the *Pv*TRAP using Kaleida graph program (Fig. 4).

**Figure 5:**
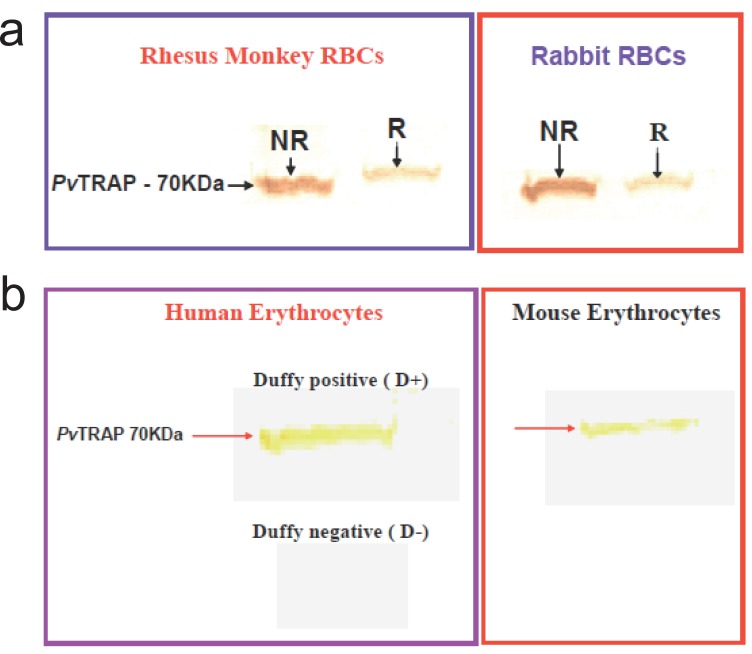
**a**. Biological and immunological characterization of purified and refolded recombinant *Pv*TRAP. Binding of purified and refolded recombinant *Pv*TRAP to rabbit and rhesus monkey erythrocytes. Erythrocytes were washed with incomplete RPMI containing 0.5 (vol.) Foetal Complement Serum. The reduced (R) and non-reduced (NR) refolded and purified *Pv*TRAP were added and incubated for 1hr at 37°C and the mixture passed through Dibutylphthalate (DBP). The protein was eluted with 1.5 M NaCl and the binding was confirmed by western blot analysis with polyclonal anti-*Pv*TRAP as the primary antibody. The left panel clearly shows the binding of both the reduced and non-reduced *Pv*TRAP to rhesus monkey erythrocytes, while the right panel indicates the binding for rabbit erythrocytes. The movement of the protein on a 10% SDS-PAGE gel under the different conditions is indicated in both cases; **b**. *Pv*TRAP binding to Human Erythrocytes: Erythrocyte binding assays were performed using Mouse and Human erythrocytes: Duffy-Positive (D+) and Duffy-Negative (D-). Purified and refolded recombinant *Pv*TRAP construct was able to bind to Duffy-Positive (D+) Human Erythrocytes, but there was no binding with Duffy-Negative (D-) Human Erythrocytes, as shown in the left panel. The right panel indicates the binding of the protein to the erythrocytes from mouse. The purified and refolded recombinant *Pv*TRAP did not bind to Human Duffy-Negative (D-) erythrocytes as shown in the present investigation.

**Erythrocyte Interaction Assay.** Purified and refolded *Pv*TRAP was further analyzed for its biological activity by employing erythrocyte binding assay according to the established protocols (32, 33). Basically Duffy-positive and Duffy-negative human erythrocytes were washed with incomplete RPMI medium. 0.5 volume of Foetal Complement Serum (FCS) was added to the washed erythrocytes with the corresponding purified and refolded *Pv*TRAP, incubated at 37°C for 1 hr. The mixture was passed through Dibutylphthalate (DBP) and the supernatant material was eluted with 1.5 M NaCl Western blot analysis was carried out on the eluted supernatant material to confirm the binding of *Pv*TRAP to the erythrocytes using *Pv*TRAP-rabbit raised antibodies to recognize the protein on the blot (Fig. 5a & b).

## RESULTS AND DISCUSSIONS

In the present investigation, expressed and purified *Pv*TRAP of about 30mg/litre recombinant protein was obtained. This showed that the nearly full length *Pv*TRAP gene (~1.4 Kb) that encoded the protein was fully expressed, as shown in Figure 3a-b. Our study revealed that the recombinant *Pv*TRAP was insoluble and present in inclusion bodies. We are reporting for the first time the expression, purification and characterization of *Pv*TRAP, even though, earlier studies on TRAP only reported the complete sequence of *P. yoelii* sporozoite surface protein 2 (SSP2) gene (34), characterization of *P. falciparum* SSP2 (6), cloning and cross species comparison of *P. knowlesi, P. vivax* and *P. gallinaceum* (9), and also sequence analysis of *P. cynomolgi* TRAP (10). In the present study, eluted recombinant *Pv*TRAP was subjected to multi-stage purification procedure, as shown in Figure 2. Single band purified, refolded *Pv*TRAP was generated (Fig. 3a) and further analyzed under different conditions (Fig. 3a & b, 4, 5a-b). Circular dichroism (CD) spectra and helical conformation of *Pv*TRAP carried out using J-720 spectropolarimeter- computer based analytical tools confirmed the conformational position of the protein at 180-260 nm. The spectrum of *Pv*TRAP recorded at pH7.2 showed a minimum at 206 nm and a shoulder at about 240 nm, typical of α-helical protein, thus, confirming that the recombinant purified *Pv*TRAP was structurally conformed and refolded (data not shown).

The binding of refolded *Pv*TRAP under different conditions, as shown in Figure 4, fully showed the capacity of this recombinant protein of binding to HepG2 liver cells. This confirmed the biological activity and structural conformation of purified and refolded *Pv*TRAP. These results suggest that *P. vivax* TRAP (*Pv*TRAP) gene which encoded the recombinant protein contained A-domain which has the properties of binding to a heparin- related ligand on HepG2 liver cells. This current observation on recombinant and refolded *Pv*TRAP firstly could be attributed to the possibility of the protein undergoing post-translational modifications in *Escherichia coli*, even though bacterial expression systems often fail to post-translationally modify a eukaryotic protein properly. Secondly, the role of A-domain earlier reported in *Plasmodium* TRAP which is present in our *Pv*TRAP construct shares certain binding properties attributed to the region II plus like domain of TRAP in all *Plasmodium* species. As shown in our study, these properties could contribute to the observed binding of *Pv*TRAP to heparin sulfate on hepatocytes. This observation clearly shows the importance of *Pv*TRAP in the pre-erythrocytic invasion of liver cells by *Plasmodium vivax*.

The level of binding of the protein to HepG2 liver cells recorded in reduced and alkylated *Pv*TRAP was comparatively less as compared to normal purified and refolded *Pv*TRAP. The refolded *Pv*TRAP had the disulfide bond structurally conformed, whereas, the reduced and alkylated *Pv*TRAP had lost the disulfide bond by treatment with DTT and Iodoacetamide. Hence, this biochemical analysis affected the protein under the reduced and alkylated conditions by changing the structural conformation as compared to normally refolded and purified *Pv*TRAP. This could be the basis for poor binding of the reduced and alkylated *Pv*TRAP to HepG2 liver cells and demonstration of the protein specificity to these cells as shown under different biochemical conditions, as well as the migration and resolution of the protein on SDS-PAGE as shown in Figure 3b and 4.

Our experimental report on *Pv*TRAP supports previous studies on binding of TRAP to sulfatide and heparin sulfate on the immortalized hepatocyte line HepG2 (14, 15, 35, 36), as well as other reported work on TRAP (37, 38). These results and the data on circular dichroism analysis on *Pv*TRAP clearly demonstrated that the purified recombinant *Pv*TRAP generated in our present investigation was properly refolded with good structural conformation, biological and functional activities.

Further analysis on the biological activity of purified and refolded *Pv*TRAP was carried out using an erythrocyte interaction assay (Fig. 5a & b). Purified and refolded recombinant *Pv*TRAP recognized and interacted with Duffy-positive (D+) human erythrocytes, while no recognition and interaction was observed with Duffy- negative (D-) erythrocytes (Fig. 5b). Positive binding was observed with Rhesus monkey, Rabbit and Mouse erythrocytes (Fig. 5a & 5b). The interaction of recombinant *Pv*TRAP with only human Duffy-positive erythrocytes is reported firstly in the present study. This data corroborated earlier findings on various studies on *Plasmodium knowlesi, Plasmodium vivax and Plasmodium cynomolgi* (41-43) and studies on binding properties of native *P. vivax* Duffy Binding protein (*Pv*DBP) and region II of TRAP protein (32, 39). Though, region II of *Pv*DBP bound to Aotus monkey erythrocytes, but no binding was reported with Rhesus monkey erythrocytes (32).

The present observation in our study revealed the interaction and recognition of *Pv*TRAP with Rhesus monkey erythrocytes. However, it is important to note that the role of TRAP as a blood stage malaria parasite potential vaccine candidate is still not clear, but *Plasmodium* TRAP is a transmembrane protein whose part of its ectodomain consists of an A-domain and a thrombospondin type 1 repeat (TSR). Earlier studies on TRAP indicated the presence of 55-60 residues in TSR, five conserved residues and metal ion – dependent adhesion site (MIDAS) motif in A-domain (15, 16-19). The TSR and A domain of TRAP specifically interact with host cell receptors during cell invasion and these interactions are used by the parasite to exert force and actively penetrate the cell.

These TRAP adhesive modules are the only parasite ligands involved in productive interactions with the cell surface during cell invasion (15), therefore, purified and refolded recombinant *Pv*TRAP which we expressed and reported here consists of these modules and they could act as a dual ligand system which appears to be important and sufficient for interaction and recognition of erythrocytes as well as potential for cell invasion (20-24). The adhesive modules present in this current protein- *Pv*TRAP, most especially metal ion-dependent adhesion site motif which was made up of five residues, could play a critical role in ligand-binding and erythrocyte adhesive properties of recombinant *Pv*TRAP. Our present observation on erythrocytic binding properties of *Pv*TRAP may seem controversial, but it has generated the focus on the need to carry out further studies on the role of *Pv*TRAP gene in erythrocytic *Plasmodium vivax* cell invasion and immuno-pathogenicity in man.

In conclusion, we have been able to express, purify and characterize refolded recombinant *Pv*TRAP. The biochemical and binding properties of the protein clearly demonstrated the potential of *Pv*TRAP as a good target and strong vivax malaria vaccine candidate.
